# Hypocellular myelodysplastic syndromes (h-MDS): from clinical description to immunological characterization in the Italian multi-center experience

**DOI:** 10.1038/s41375-022-01592-3

**Published:** 2022-05-21

**Authors:** Giulia Calabretto, Enrico Attardi, Antonella Teramo, Valentina Trimarco, Samuela Carraro, Sandra Mossuto, Gregorio Barilà, Cristina Vicenzetto, Vanessa Rebecca Gasparini, Monica Crugnola, Pasquale Niscola, Antonella Poloni, Valentina Giai, Valentina Gaidano, Carlo Finelli, Roberta Bertorelle, Cinzia Candiotto, Marco Pizzi, Gianni Binotto, Monica Facco, Fabrizio Vianello, Livio Trentin, Gianpietro Semenzato, Renato Zambello, Valeria Santini

**Affiliations:** 1 Hematology Division, Department of Medicine, Padua University School of Medicine, Padua, Italy; 2 Veneto Institute of Molecular Medicine (VIMM), Padua, Italy; 3 MDS Unit, Division of Hematology, AOU Careggi-University of Florence, Florence, Italy; 4 Italian MDS Foundation (FISIM - ETS), Bologna, Italy; 5 Hematology Unit, Ospedale dell’Angelo, Venezia-Mestre, Italy; 6 Hematology Unit and BMT Center, AOU-Parma, Parma, Italy; 7 Department of Hematology, S. Eugenio Hospital, Rome, Italy; 8 Division of Hematology, AOU Ospedali Riuniti - Università Politecnica Marche, Ancona, Italy; 9 Division of Hematology, Department of Oncology, AOU Città della Salute e della Scienza, Turin, Italy; 10 Department of Hematology, AO SS Antonio e Biagio e Cesare Arrigo, Alessandria, Italy; 11 IRCCS Azienda Ospedaliero-Universitaria di Bologna, Istituto di Ematologia “Seràgnoli”, Bologna, Italy; 12 Immunology and Molecular Oncology Unit, Veneto Institute of Oncology IOV-IRCCS, Padua, Italy; 13 Department of Medicine, Surgical Pathology and Cytopathology Unit, University School of Medicine, Padua, Italy

**Keywords:** Myelodysplastic syndrome, Lymphocytes

**TO THE EDITOR:**

Myelodysplastic Syndromes (MDS) are a highly heterogeneous group of blood neoplasias characterized by myeloid dysplasia, ineffective hematopoiesis and increased risk of progression to acute myeloid leukemia [1]. We focused on hypocellular-MDS (h-MDS), a rare subtype accounting for 10–15% of MDS patients, that is defined by an age-adjusted reduction of bone marrow (BM) cellularity or, according to Aplastic Anemia definition, by a BM cellularity <30% [2].

Although the WHO classification of myeloid neoplasms and acute leukemia does not recognize h-MDS as a distinct entity, these patients are typically younger and characterized by more severe cytopenias, higher transfusion dependence and lower blast percentages as compared to normo/hypercellular MDS (n-MDS) [3–5]. Data on h-MDS outcome, instead, are still inconsistent [4, 6]. Beside these clinical features, h-MDS share molecular characteristics, including karyotype abnormalities, that suggest a common underlying pathogenesis [4, 5].

A barely understood biology, ill-defined diagnostic criteria and the lack of conclusive prognostic data hindered the establishment of specific treatment guidelines for h-MDS. The evaluation of clinical outcome following immunosuppressive therapy (IST) in MDS patients indicated peculiar responses in the hypoplastic category [7, 8], supporting a pathogenetic role of immune system alterations. In this regard, the immunological characterization of h-MDS should help to improve the risk stratification of patients and choice of therapy.

Taking advantage of the National Registry of the Italian Foundation of MDS (FISiM), we evaluated clinical features, overall survival (OS) and treatment of h-MDS in comparison with n-MDS. A cohort of 1945 MDS patients, enrolled in the FISiM registry, was included in the study. Patients were selected based on the availability of bone trephine biopsy evaluation and complete clinical annotations. Diagnosis of h-MDS was assumed for BM cellularity **≤**30% and the prognostic stratification of patients was defined according to the Revised International Prognostic Scoring System (IPSS-R) [9].

Within the main cohort, 336/1945 (17%) patients were recognized as h-MDS and 1609/1945 (83%) as n-MDS. The age of patients ranged between 18 to 106 years, with a global M/F ratio of 1.56. According to BM cellularity, median age was 75 and 74 years in the h-MDS and n-MDS groups, respectively; the M/F ratio was 1.14 in h-MDS and 1.67 in n-MDS (*p* < 0.01).

The stratification of patients into IPSS-R risk categories was similar between h-MDS and n-MDS (Fig. 1A). An IPSS-R score of 3.5 was used to stratify patients into two main groups: low-risk IPSS-R (LR, score ≤3.5) and high-risk IPSS-R (HR, score >3.5). Accordingly, 271/336 (81%) h-MDS were placed in the LR and 65/336 (19%) in the HR categories; similarly, 1176/1609 (73%) of n-MDS were included in the LR, while 433/1609 (27%) in the HR groups.

**Fig. 1:**
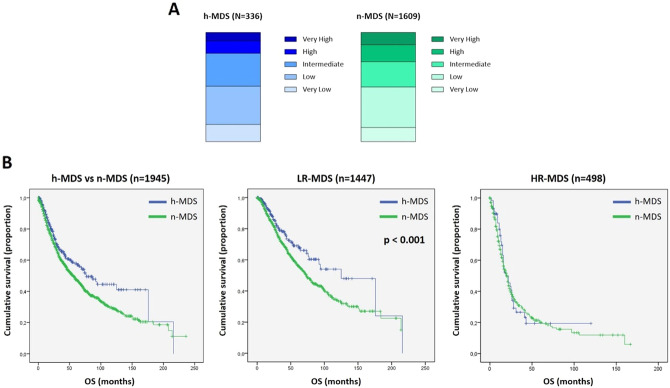
Risk stratification and OS of h-MDS compared to n-MDS. **1-A** Patients stratification into IPSS-R risk categories. H-MDS are represented in shades of blue (with light blue stating for very low risk and dark blue stating for very high risk categories); n-MDS are represented in shades of green (with light green stating for very low risk and dark green stating for very high risk categories). In detail, h-MDS were distributed as follows: 15.5% Very Low, 35.1% Low, 30.1% Intermediate, 11.3% High, 8% Very High. N-MDS patients were classified as: 12.8% Very Low, 37.2% Low, 23.1% intermediate, 15.5% High, 11.4% Very High. **1-B** Kaplan–Meier curves showing OS in the entire MDS cohort (left panel), LR-MDS (central panel) and HR-MDS (right panel), comparing h-MDS (in blue) vs n-MDS (in green). Vertical lines denote censored patients. Median OS is significantly higher in LR h-MDS as compared to LR n-MDS (central panel, 125 vs 74 months, *p* < 0.001). Note: h-MDS, hypocellular myelodysplastic syndromes; HR high-risk; LR low-risk; n-MDS normo-/hypercellular myelodysplastic syndromes; OS overall survival.

Median OS was 77 months for h-MDS and 56 months for n-MDS (*p* > 0.05). According to IPSS-R stratification and BM cellularity, LR h-MDS had a median OS of 125 months, while LR n-MDS of 74 months (*p* < 0.001). Conversely, median OS in HR MDS was comparable, i.e. 19 months in HR h-MDS patients vs 20 months in n-MDS cases (*p* > 0.05) (Fig. 1B).

Beyond its clinical relevance, our observations provide reliable data on h-MDS outcome, possibly clarifying previously reported inconsistencies [4, 10]. Most importantly, this evidence suggests that discrete biological features might account for the divergent course of the disease.

MDS are known to be characterized by a wide spectrum of immunological deregulations [1], including the frequent expansion of LGL clones, reaching the diagnostic criteria of LGL Leukemia (LGLL) in up to 27% of cases [11, 12]. This prompted us to better characterize the immunological landscape of h-MDS patients, at the time of diagnosis, focusing on cytotoxic T and NK cell subsets.

A restricted subgroup of 12 h-MDS patients was enrolled within the FISiM-hMDS14 sub-study (Supplementary Table 1), to investigate the immune mechanisms in this peculiar disease subset. The study was approved by local ethic committees and patients signed informed consent, according to the Helsinki Declaration.

Immunophenotypic analysis was performed on both peripheral blood (PB) and BM samples. A CD3+/CD4−/CD8+/CD16±/CD56−/CD57+cytotoxic T-LGL expansion was found in 8/12 (66%) cases in PB samples (range: 14–46% of lymphocytes) and in all the BM samples (range: 7–46% of lymphocytes), with respect to normal values (range: 6 ± 3% and 4 ± 2% of lymphocytes in PB and BM, respectively) (Supplementary Table 2). No recurrent TCR-Vβ immunodominant expansions were observed. To distinguish clonal from reactive expansions, TCR rearrangement was evaluated on DNA from PB and BM mononuclear cells (PBMC and BMMC). TCR clonality was demonstrated in 6/12 (50%) patients, with a concordance between PB and BM (Supplementary Table 2). Noteworthy, the threshold commonly accepted for LGLL diagnosis (LGL > 0.5 × 10^9^/L) was reached only in 2/6 (33%) cases with a T cell clone, i.e. in the 17% of the h-MDS subgroup.

The immunophenotypic characterization of NK cell compartment showed CD3−/CD16^bright^/CD56^dim/neg^ NK cell expansions in 4/12 (33%) h-MDS cases (range: 19–30% and 19–22% of lymphocytes in PB and BM, respectively), as compared to physiologic condition (range: 13 ± 5% and 6 ± 5% of lymphocytes in PB and BM, respectively) (Supplementary Table 2). NK cells were characterized by an effector-memory phenotype, based on the expression of CD57 and lack of CD62L. Evaluation of Killer Immunoglobulin-like receptors (KIR) confirmed a restricted pattern of expression, characterized by the prevalent expression of CD158b (i.e. KIR2DL2/L3) in all the 4 cases (range: 70–78% of CD3-CD16 + NK cells); of these, 3/4 (75%) were also characterized by the expression of the activating NKG2C receptor (Supplementary Table 2). As for clonal T cell proliferations, KIR restrictions in NK cell populations were detected both in PB and BM samples.

In line with previous reports, we observed that the immunological landscape of h-MDS patients is characterized by T and NK cell expansions [5, 13]. The novelty of our findings relies on the dominant involvement of different LGL subsets, according to patient prognostic stratification. In detail, 5/6 (83%) patients with a T-cell clone were included among the HR group, while only 1/6 (17%) was placed in the LR category. Conversely, 3/4 (75%) patients with a NK cell clonal expansion were included in the LR group, while 1/4 (25%) fell in the HR category (Fig. 2). Although our findings need to be confirmed in a larger number of cases, the observed immunological differences could be at the basis of the improved OS that we exclusively observed in the LR h-MDS group.

**Fig. 2:**
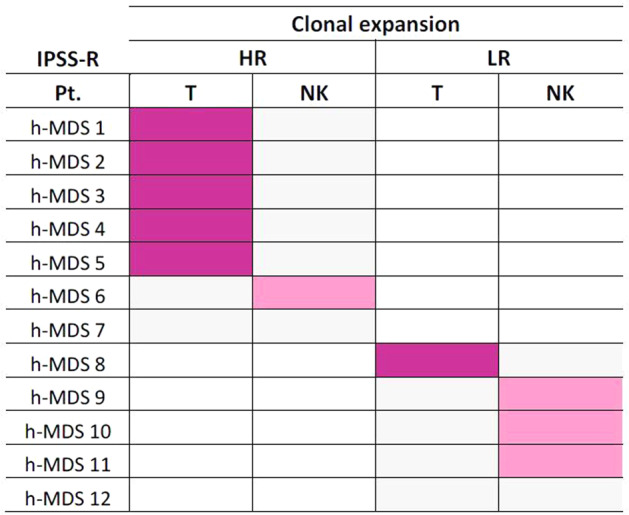
Distribution of T cell (purple) and NK cell (pink) clonal populations in h-MDS patients (*n* = 12) according to the IPSS-R stratifications. Clonality of T and NK cell expansions was assessed in a cohort of 12 h-MDS patients enrolled within the FISiM-hMDS14 sub-study and data were evaluated according to patients IPSS-R. T cell clonal expansions are represented in purple, while NK cell clonal expansions are represented in pink. A peculiar associations of T-cell clones with HR h-MDS and of clonal NK cell expansions with LR h-MDS was observed. No contemporary detection of clonal T and NK cell populations was found. In two patient (#7 and #12) any clonality was detected. Note: h-MDS, hypocellular myelodysplastic syndromes; HR higher risk; IPSS-R Revised International Prognostic Scoring System; LR lower risk; NK natural killer.

We previously reported the coexistence of T cell clones in ~50% of patients with chronic lymphoproliferative disorder of NK cells (CLPD-NK) [14]. In h-MDS patients, instead, no contemporary detection of TCR or KIR restriction was observed (Fig. 2), suggesting that T and NK clonal expansions may be mutually exclusive. This different involvement of T or NK cell subsets hints at distinct functional events taking place along h-MDS course, reminding the impairment of NK cell activity involved in n-MDS progression [1].

At a molecular level, we observed the presence of *STAT3* activating mutations in PBMC and BMMC of 2/12 (17%) h-MDS cases (Supplementary Table 1). *STAT3* mutated patients were those who fulfilled the diagnostic criteria of LGLL and they were both characterized by a monoclonal TCRɑβ/CD3+/CD4−/CD8+/CD16+/CD56−/CD57+T-LGL expansion and included in the HR group. Based on this evidence, *STAT3* mutations might be associated with a worse prognosis with respect to *STAT3* wild-type cases, as in LGLL patients [15]. Consistently, *STAT3* mutated T cytotoxic clones may promote a chronic inflammatory BM environment and a persistent deregulated immune activation, leading to disease progression in a discrete subset of h-MDS cases.

In line with this consideration, different hypotheses have been proposed to explain the peculiar association between LGL and myeloid clones [11]. This is quite rare, possibly representing an extreme condition caused by common age-related pathogenetic mechanisms (i.e. a pro-inflammatory environment and mutational stress). Otherwise, LGLL may evolve from an immune surveillance reaction, with aberrant hematopoietic stem cells (HSC) triggering LGL activation and clonal expansion; on the contrary, clonal LGL might themselves promote a damage in the HSC compartment, leading to MDS development.

Beyond their pathogenetic role, the observed immune alterations may have relevant clinical implications, supporting the rationale for the administration of immunosuppressive agents. In our cohort, we observed that IST is rarely applied, irrespective of BM cellularity. In detail, immunosuppressive treatments were employed for 0.4% and 1.2% of LR h-MDS and n-MDS, respectively (Supplementary Table 3). Notwithstanding, our results in h-MDS suggest that treatment with immunosuppressive agents could be an effective strategy in this disease subset. Most importantly, the immunological features of h-MDS patients may be involved in the mechanism and duration of treatment responses.

In conclusion, we report an unbiased clinical analysis of the FISiM registry, based on the largest series of h-MDS (336 patients) so far evaluated, in comparison with an extended cohort of n-MDS. Of note, we showed a significant longer OS in LR h-MDS vs LR n-MDS.

In a preliminary investigation of h-MDS patients, combining phenotypic and molecular analyses, the LR group resulted to be characterized by KIR/NKG2 restricted NK cell expansions, whereas HR h-MDS were associated with T cell clones. Prospective studies are ongoing to better define the prognostic roles of the different LGL subsets in these patients. Remarkably, our observations might pave the way for the establishment of prospective trials to evaluate the efficacy of IST in h-MDS patients and the modulation of their T and NK cell repertoire.

## Supplementary information

Supplemental material

Supplemental material

## References

[1] Cazzola M (2020). Myelodysplastic Syndromes. N. Engl J Med.

[2] Hasserjian RP, Orazi A, Brunning R, Germing U, Le Beau MM, Porwit A, et al. Myelodysplastic syndromes: Overview. In: Swerdlow SHCE, Harris NL, Jaffe ES, Pileri SA, Stein H, Thiele J, et al. WHO Classification of Tumors Of Haematopoietic and Lymphoid Tissues. Lyon (France): IARC; 2017. 98–106.

[3] Huang T-C, Ko B-S, Tang J-L, Hsu C, Chen C-Y, Tsay W (2008). Comparison of hypoplastic myelodysplastic syndrome (MDS) with normo-/hypercellular MDS by International Prognostic Scoring System, cytogenetic and genetic studies. Leukemia.

[4] Bono E, McLornan D, Travaglino E, Gandhi S, Gallì A, Khan AA (2019). Clinical, histopathological and molecular characterization of hypoplastic myelodysplastic syndrome. Leukemia.

[5] Fattizzo B, Serpenti F, Barcellini W, Caprioli C (2021). Hypoplastic myelodysplastic syndromes: just an overlap syndrome?. Cancers.

[6] Tong W-G, Quintás-Cardama A, Kadia T, Borthakur G, Jabbour E, Ravandi F (2012). Predicting survival of patients with hypocellular myelodysplastic syndrome: development of a disease-specific prognostic score system. Cancer.

[7] Stahl M, DeVeaux M, de Witte T, Neukirchen J, Sekeres MA, Brunner AM (2018). The use of immunosuppressive therapy in MDS: clinical outcomes and their predictors in a large international patient cohort. Blood Adv.

[8] Stahl M, Bewersdorf JP, Giri S, Wang R, Zeidan AM (2020). Use of immunosuppressive therapy for management of myelodysplastic syndromes: a systematic review and meta-analysis. Haematologica.

[9] Greenberg PL, Tuechler H, Schanz J, Sanz G, Garcia-Manero G, Solé F (2012). Revised international prognostic scoring system for myelodysplastic syndromes. Blood.

[10] Sloand EM (2009). Hypocellular myelodysplasia. Hematol Oncol Clin North Am.

[11] Durrani J, Awada H, Kishtagari A, Visconte V, Kerr C, Adema V (2020). Large granular lymphocytic leukemia coexists with myeloid clones and myelodysplastic syndrome. Leukemia.

[12] Komrokji RS, Ali NA, Sallman D, Padron E, Lancet J, Sokol L (2020). Characterization of myelodysplastic syndromes (MDS) with T-cell large granular lymphocyte proliferations (LGL). Leukemia.

[13] Karantanos T, DeZern AE (2021). Biology and clinical management of hypoplastic MDS: MDS as a bone marrow failure syndrome. Best Pract Res Clin Haematol.

[14] Gattazzo C, Teramo A, Passeri F, De March E, Carraro S, Trimarco V (2014). Detection of monoclonal T populations in patients with KIR-restricted chronic lymphoproliferative disorder of NK cells. Haematologica.

[15] Barilà G, Teramo A, Calabretto G, Vicenzetto C, Gasparini VR, Pavan L (2020). Stat3 mutations impact on overall survival in large granular lymphocyte leukemia: a single-center experience of 205 patients. Leukemia.

