# Supplementary material for: Hypocellular myelodysplastic syndromes (h-MDS): from clinical description to immunological characterization in the Italian multi-center experience
Source: Leukemia. 2022 May 21;36(7):1947–50. doi: 10.1038/s41375-022-01592-3 (PMC9252912; doi:10.1038/s41375-022-01592-3)
Supplement: Supplementary file 1 — Supplemental material [file 41375_2022_1592_MOESM1_ESM.pdf]

Supplementary Table 1. Clinical and molecular features of h-MDS patients (N=12, enrolled in the FISiM-hMDS14 sub-study) at the diagnosis.

PATIENT	AGE/ SEX	IPSS-R	Hb	MCV	ANC	PLT	Blasts BM (%)	STAT3* MUT	MYELOID MUT
h-MDS 1	64/F	HR	9.7	114.9	0.33	78	<2%	WT	ND
h-MDS 2	82/F	HR	8.7	ND	1.59	7	<2%	WT	*JAK2*
h-MDS 3	84/M	HR	9.6	112.3	0.80	100	<2%	N647I	ND
h-MDS 4	85/F	HR	9.9	96.1	0.26	136	2-4%	Y640F	ND
h-MDS 5	97/F	HR	9.9	116.3	1.4	23	<5%	WT	ND
h-MDS 6	66/ND	HR	9.0	112	1.02	188	5-10%	WT	*SF3B1, TET2*
h-MDS 7	81/M	HR	8.7	97	ND	10	<2%	WT	*DNMT3A*
h-MDS 8	42/M	LR	11.4	109	ND	83	<2%	WT	*CEBPA*
h-MDS 9	38/F	LR	10.9	87.8	2.4	141	2-4%	WT	*CEBPA, DNMT3A*
h-MDS 10	80/F	LR	11.6	97	1.23	126	<2%	WT	ND
h-MDS 11	89/ND	LR	11.5	102	1.10	106	<2%	WT	ND
h-MDS 12	64/F	LR	12.8	105	1.07	85	<2%	WT	*DNMT3A*

*All patients were wild-type in STAT5b (exons 16-18).

Note: ANC, absolute neutrophil count; BM, bone marrow; F, female; Hb, hemoglobin; HR, higher risk; IPSS-R, Revised International Prognostic Scoring System; LR, lower risk; M, male; MUT, mutations; ND, not determined; PB, peripheral blood; PLT, platelets; Pt, patient.

Supplementary Table 2. Immunophenotypic and clonality evaluation of h-MDS (N=12) with respect to healthy controls (N=25)

	PB					BM					TCR REARRANGMENT	KIR - NKG2 RESTRICTION*
	CD3+CD4+ (% on Ly)	CD3+CD8+ (% on Ly)	CD3+CD57+ (% on Ly)	CD3-CD16+ (% on Ly)	CD3-CD56+ (% on Ly)	CD3+CD4+ (% on Ly)	CD3+CD8+ (% on Ly)	CD3+CD57+ (% on Ly)	CD3-CD16+ (% on Ly)	CD3-CD56+ (% on Ly)		
**h-MDS 1**	46	43	26	3	7	40	45	25	2	3	Clonal	NO
**h-MDS 2**	11	65	25	12	20	17	61	24	5	5	Clonal	NO
**h-MDS 3**	26	68	30	2	1	16	75	24	1	1	Clonal	NO
**h-MDS 4**	40	54	46	3	1	30	61	46	2	1	Clonal	NO
**h-MDS 5**	44	25	16	2	3	42	28	14	5	5	Clonal	NO
**h-MDS 6**	49	29	8	19	22	31	36	11	22	21	Policlonal	CD158B, NKG2C
**h-MDS 7**	61	20	7	8	13	55	29	7	5	10	Policlonal	NO
**h-MDS 8**	35	45	22	6	10	30	48	28	10	11	Clonal	NO
**h-MDS 9**	43	24	8	21	20	40	26	8	20	21	Policlonal	CD158B
**h-MDS 10**	41	39	14	29	30	47	34	15	19	19	Policlonal	CD158B, NKG2C
**h-MDS 11**	32	38	14	23	21	20	44	13	22	19	Policlonal	CD158B, NKG2C
**h-MDS 12**	71	18	6	2	3	46	34	16	7	8	Policlonal	NO
**CTR (N=25) range values**	47% ± 7	30% ± 7	6% ± 3	13% ± 5	13% ± 5	31% ± 7	28% ± 7	4% ± 2	6% ± 5	6% ± 5	Policlonal	NO

* KIR: a restricted pattern of Killer Immunoglobulin-like receptors (KIR) include the dominant expression of a relevant KIR or the lack of KIR expression, as compared to healthy controls (CTR). Normal values, referred to the gate of CD3-CD16+ lymphocytes, are: CD158a: 20%±10; CD158b: 30%±10; CD158e: 13%±5; NKG2A: 45%±20; NKG2C: <5%.

Note: BM, bone marrow; CD, cluster of differentiation; CTR, healthy controls; KIR, Killer Immunoglobulin-like receptor; h-MDS, hypoplastic Myelodysplastic syndrome; Ly, lymphocytes; N, number; PB, peripheral blood; TCR, T cell Receptor.

Supplementary Table 3. Patients and first line therapies for h-MDS in comparison with n-MDS patients.

**MDS (N)**	**h-MDS (336)**		**n-MDS (1609)**	
**BM Cellularity**	≤ 30%		> 30%	
**Age (median)**	75		74	
**Sex (M/F)**	1.14		1.67	
**IPSS-R (N, %)**	**LR (271, 80.7)**	**HR (65, 19.3)**	**LR (1176, 73.1)**	**HR (433, 26.9)**
**Therapy (%)**				
Observation/BSC	33.8	12.1	31.6	16.1
ESA	42.6	29.3	41.2	24.8
Lenalidomide	3.8	0	0.7	0.5
Differentiation therapy (ATRA)	4.6	5.2	6.4	7.9
**IST**	**0.4**	**0**	**1.2**	**0.5**
Azacitidine	5.1	36.2	2.8	25.1
Low-dose Chemotherapy	0	0	4.7	5.0
AML-Like Chemotherapy	0.4	1.7	0.3	6.2
HSCT	0	1.7	0.1	1.0
Experimental trials	1.7	5.2	3.9	6.2
Others	7.6	8.6	7.1	6.7

Note: AML, acute myeloid leukemia; ATRA, All-trans retinoic acid; BM, bone marrow; BSC, best supportive care; ESA, Erythroid stimulating agents; F, female; h-MDS, hypocellular myelodysplastic syndromes; HR, higher risk; HSCT, hematopoietic stem cell transplant; IPSS-R, Revised International Prognostic Scoring System; IST, immunosuppressive therapy; LR, lower risk; M, male; N, number; n-MDS, normo-/hypercellular myelodysplastic syndromes;
