# Supplementary material for: Hypocellular myelodysplastic syndromes (h-MDS): from clinical description to immunological characterization in the Italian multi-center experience
Source: Leukemia. 2022 May 21;36(7):1947–50. doi: 10.1038/s41375-022-01592-3 (PMC9252912; doi:10.1038/s41375-022-01592-3)
Supplement: Supplementary file 2 — Supplemental material [file 41375_2022_1592_MOESM2_ESM.docx]

**MATERIAL AND METHODS**

I**mmunophenotypic analysis**

Immunophenotypic analysis was performed on EDTA-treated PB and BM samples of h-MDS patients. The expression of T and NK cell markers and receptors was analyzed on PB and BM mononuclear cells (PBMC, BMMC), isolated by Ficoll-Hypaque gradient centrifugation. Cells were stained with commercially available monoclonal antibodies (mAb), including CD3 (SK7), CD4 (RPA-T4), CD8 (RPA-T8), CD16 (B73.1), CD56 (B159) and CD57 (NK-1) (Becton Dickinson). The T cell Receptor Variable β-chain Region (TCR-Vβ) of T-LGL was determined with the IOTest Beta Mark TCR-Vβ Repertoire kit (Beckman Coulter), covering approximately 70% of the normal human TCR-Vβ repertoire. KIR and CD94-NKG2A/NKG2C expression was evaluated using CD158a (Hp-3E4), CD158e (DX9) (BD Biosciences), CD158b (CH-L), CD94 (HP-3D9) (BD Pharmingen), NKG2A (131411) and NKG2C (134591) (R&D Systems) mAb. Data were acquired with the FACSCanto II and analyzed with the FACSDiva software program (BD Biosciences).

The presence of a T cell expansion was defined according to the physiologic range for CD3+CD57+ T cell, i.e. 6%±3 in PB and 4%±2 in BM samples. Characterization of NK cell subsets was performed as previously reported(1).

**Clonality assessment**

Clonal T cell expansions were determined by standard fragment length analysis of TCR-γ gene rearrangement on PBMC and BMMC samples of h-MDS patients, with the IdentiClone TCRγ gene Rearrangement Assay (Invivoscribe) and the ABI 3130 genetic analyzer (Applied Biosystem). Sequencing results were analyzed with the Peak Scan software (Invivoscribe). A restricted pattern of KIR expression, evaluated by flow cytometry, was used as a surrogate marker of NK cell clonal expansion.

**Mutation analysis**

DNA from PBMC and BMMC of patients was extracted with the Gentra Puregene Cell Kit Plus (Qiagen). Mutations in *STAT3* and *STAT5b* were analyzed by Sanger sequencing with primers covering the hotspot regions of *STAT3* (exons 19–21)(2) and *STAT5b* (exons 16-18)(3). Purified PCR products were sequenced using dye terminator technology and an ABI 3130 sequencer (Applied Biosystem); sequencing results were analyzed with the ChromasPro software. In addition, Amplification Refractory Mutation System (ARMS) polymerase chain reaction (PCR) was performed to investigate the most frequent *STAT3* variants (*i.e.* Y640F and D661Y), as previously reported(28).

DNA from BM samples was also screened for myeloid mutations by NGS, with a panel covering the most frequently mutated genes in MDS (*ABL1, BRAF, CALR, CBL, CEBPA, CSF3R, ETV6, FLT3, HRAS, IDH1, IDH2, JAK2, KIT, KRAS, MPL, NPM1, NRAS, PTPN11, RUNX1, SETBP1, SRSF2, TP53, U2AF1, WT1, ZRSR2, TET2, DNMT3A, ASXL1, EZH2, SF3B1*) by Myeloid Solution (Sophia Genetics) and a MiSeq Illumina platform.

**Statistical analysis**

Demographics and clinical characteristics of patients were summarized as median (range) for continuous variables and as numbers (%) for categorical variables. OS was defined as the time from diagnosis to death or last follow-up evaluation. Time-to-event endpoints were analyzed using the Kaplan–Meier method. A two‐tailed P‐value<0.05 was considered statistically significant. All analyses were performed using SPSS software.

**REFERENCES**

1 [Barilà G, Teramo A, Calabretto G, Ercolin C, Boscaro E, Trimarco V *et al.* Dominant cytotoxic NK cell subset within CLPD-NK patients identifies a more aggressive NK cell proliferation. *Blood Cancer J* 2018; **8**: 51.](http://paperpile.com/b/M3oNlt/S28D)

2 [Koskela HLM, Eldfors S, Ellonen P, van Adrichem AJ, Kuusanmäki H, Andersson EI *et al.* Somatic STAT3 mutations in large granular lymphocytic leukemia. *N Engl J Med* 2012; **366**: 1905–1913.](http://paperpile.com/b/M3oNlt/7mpJ)

3 [Rajala HLM, Eldfors S, Kuusanmäki H, Andersson EI, van Adrichem AJ, Lagström S *et al.* Discovery of STAT5b Mutations and Small Subclones of STAT3 Mutations in Large Granular Lymphocytic (LGL) Leukemia. Blood. 2012; **120**: 871–871.](http://paperpile.com/b/M3oNlt/Jq31)
